# Supplementary material for: ADRA2A and IRX1 are putative risk genes for Raynaud’s phenomenon
Source: Nat Commun. 2023 Oct 12;14:6156. doi: 10.1038/s41467-023-41876-5 (PMC10570309; doi:10.1038/s41467-023-41876-5)
Supplement: Supplementary file 1 — Supplementary Information [file 41467_2023_41876_MOESM1_ESM.pdf]

Supplementary Tables and Figures

ADRA2A and *IRX1* are putative risk genes for Raynaud's phenomenon

Sylvia Hartmann[1], Summaira Yasmeen[1], Benjamin M Jacobs[2], Spiros Denaxas[3,4,5,6], Munir Pirmohamed[7], Eric R. Gamazon[8], Mark J. Caulfield[9], Genes & Health Research Team, Harry Hemingway[3,4,6], Maik Pietzner[1,10,11*], Claudia Langenberg[1,10,11,*]

[1]Computational Medicine, Berlin Institute of Health at Charité – Universitätsmedizin Berlin, Berlin, Germany
[2]Preventive Neurology Unit, Wolfson Institute of Population Health, Queen Mary University of London, London, UK
[3]Institute of Health Informatics, University College London, London, UK
[4]Health Data Research UK, London, UK
[5]British Heart Foundation Data Science Centre, London, UK
[6]National Institute of Health Research University College London Hospitals Biomedical Research Centre
[7]Department of Pharmacology and Therapeutics, The Wolfson Centre for Personalised Medicine, University Liverpool, Liverpool, UK, Liverpool, UK
[8]Vanderbilt Genetics Institute, Vanderbilt University Medical Center, Nashville, TN 37203, USA
[9]William Harvey Research Institute, Queen Mary University of London, London, UK
[10]MRC Epidemiology Unit, University of Cambridge, Cambridge, UK
[11]Precision Healthcare Institute, Queen Mary University of London, London, UK
*these authors jointly supervised the work

Correspondence

Prof Claudia Langenberg (Claudia.langenberg@bih-charite.de)
Dr Maik Pietzner (maik.pietzner@bih-charite.de)

Content

Supplementary Figures 2
- Supplementary Figure 1 – Inclusion criteria and primary RP definition 2
- Supplementary Figure2 – Regional association plot 3
- Supplementary Figure 3 – Q-Q plot 4
- Supplementary Figure 4 – Beta-correlation plot 5
- Supplementary Figure 5 – Genetic correlation plot 6

Supplementary Tables 7
- Supplementary Table 1 – Characteristics of cases and controls 7
- Supplementary Table 2 – OR for sex and sex*SNP interaction 8
- Supplementary Table 3 – Effect estimates for all and primary RP cases 9
- Supplementary Table 6b – Latent causal variable analysis 10

Supplementary Figures

Supplementary Figure 1 – Inclusion criteria and primary RP definition

Flowchart for the criteria for inclusion into the study and criteria for definition as primary Raynaud's phenomenon.

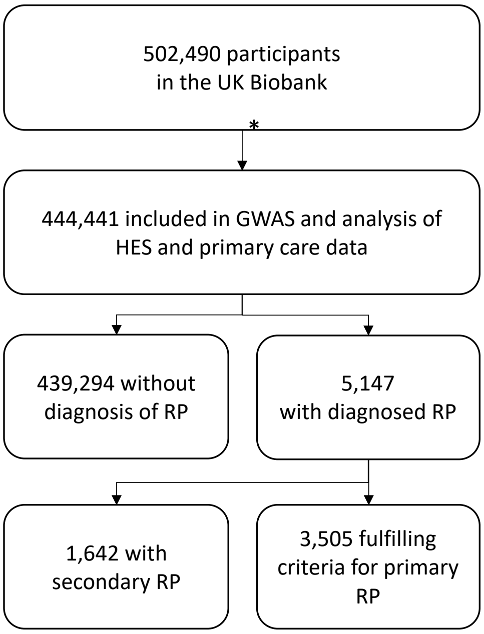

Criteria for primary Raynaud Phenomenon:

Prior and later to the diagnosis of RP the following diseases have never been diagnosed:

- Arteriosclerosis
- Dermatomyositis
- Polymyositis
- Systemic sclerosis
- Systemic Lupus Erythematosus
- Sjoegren Syndrome
- Diseases of connective tissue
- Cryoglobulinemia
- Autoimmune haemolytic anaemias
- Carpal tunnel syndrome
- Frostbite
- Hypothyroidism
- Medication with beta blockers

RP = Raynaud phenomenon, HES = hospital episode statistics
*Excluded due to missing genetic data, genetic data did not pass quality control, or withdrawn consent

Supplementary Figure2 – Regional association plot

Regional association plot for variants reported in table 1 for Raynaud's Phenomenon (RP). Each plot displays p-values from logistic regression models associating single nucleotide variants in a ±500kb window around the lead signal with the risk of RP. The colour gradient indicates the linkage disequilibrium (r^2) with the lead signal (annotated) at the respective locus.

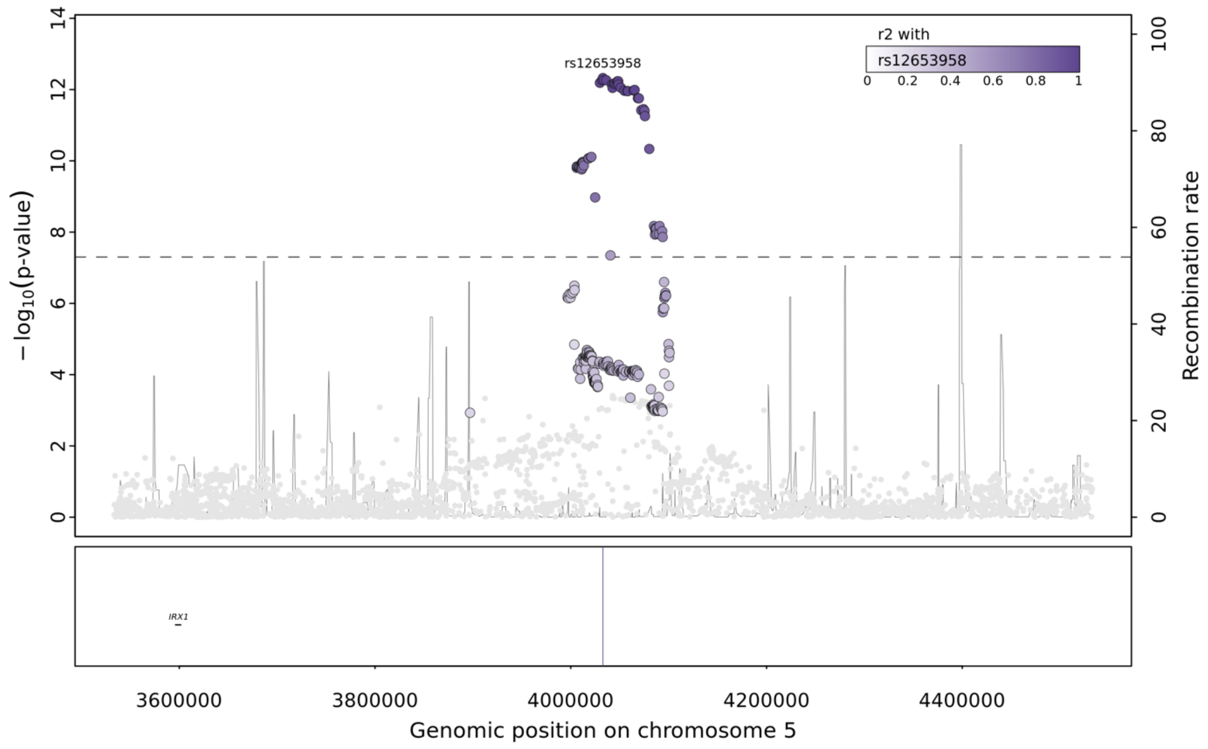

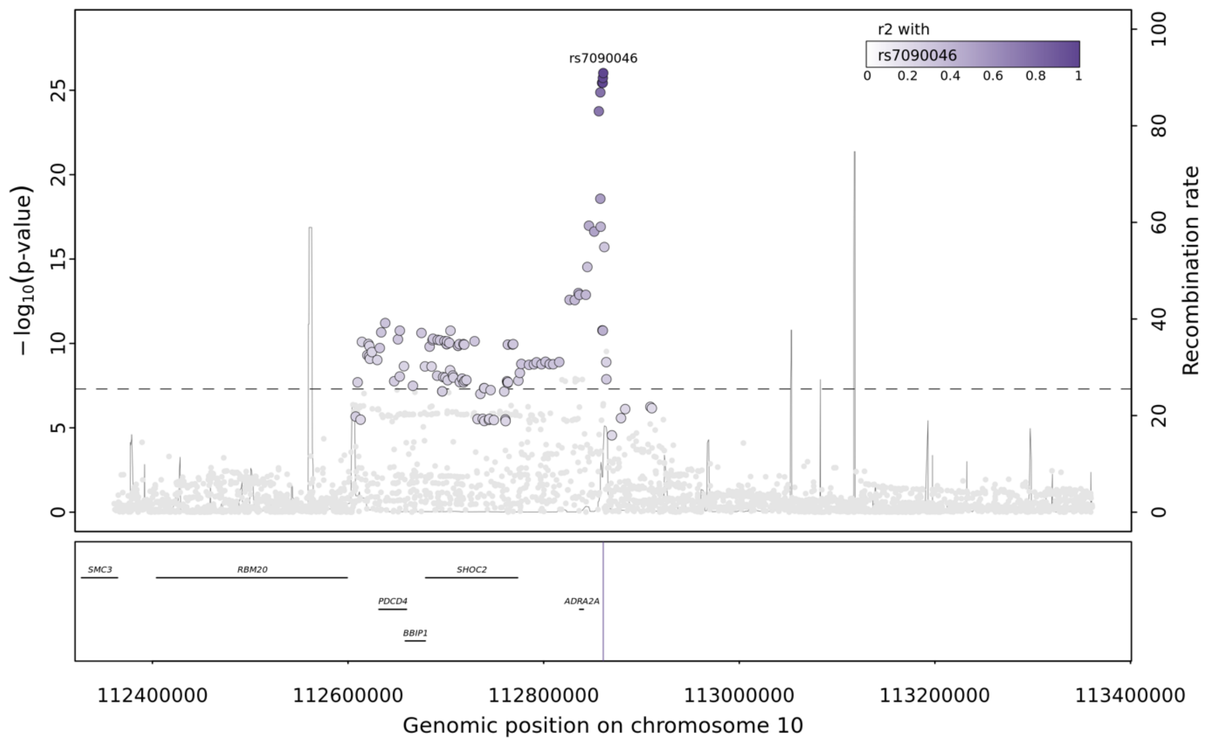

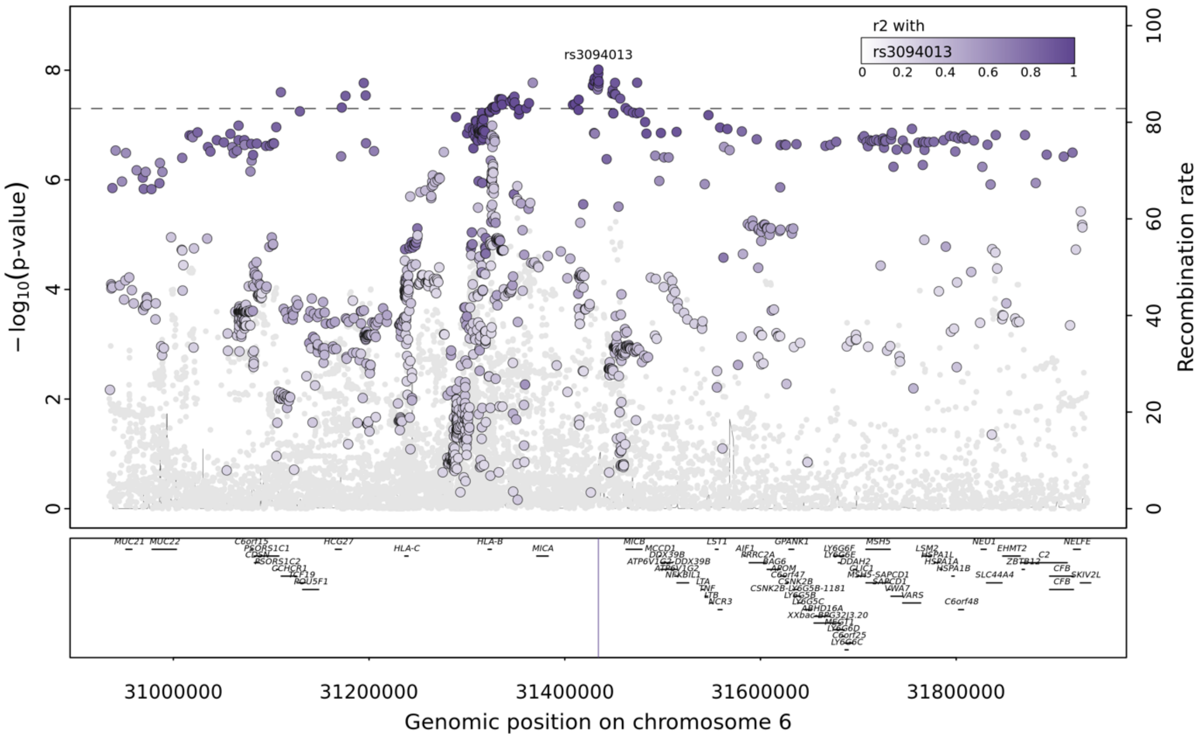

Supplementary Figure 3 – Q-Q plot

Q-Q plot

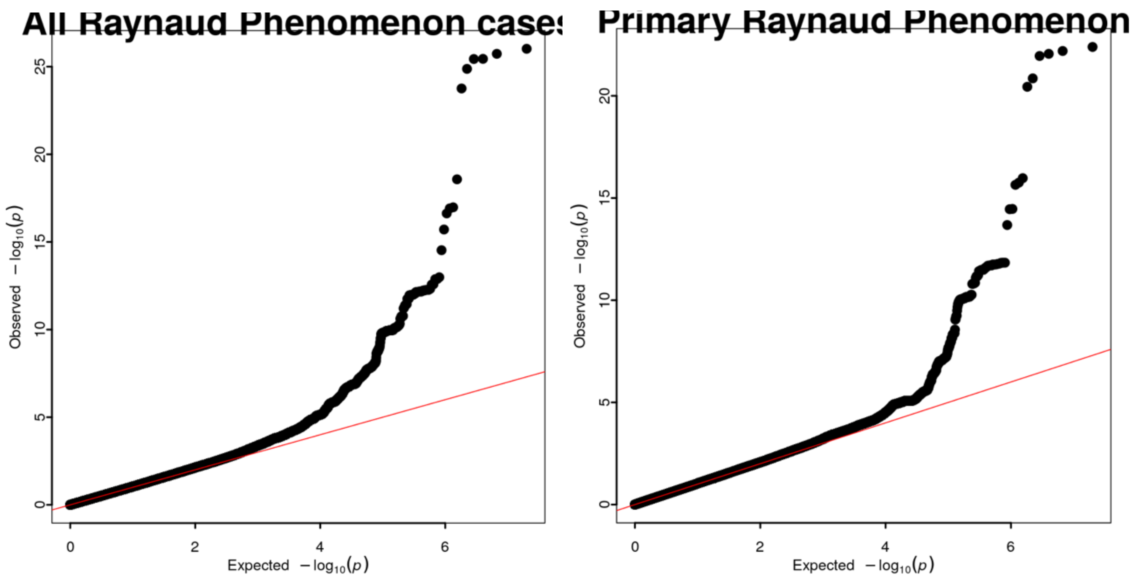

Supplementary Figure 3 QQ-plot of the genome-wide association results for all RP cases (left) and restricting to primary RP cases (right).

Supplementary Figure 4 – Beta-correlation plot

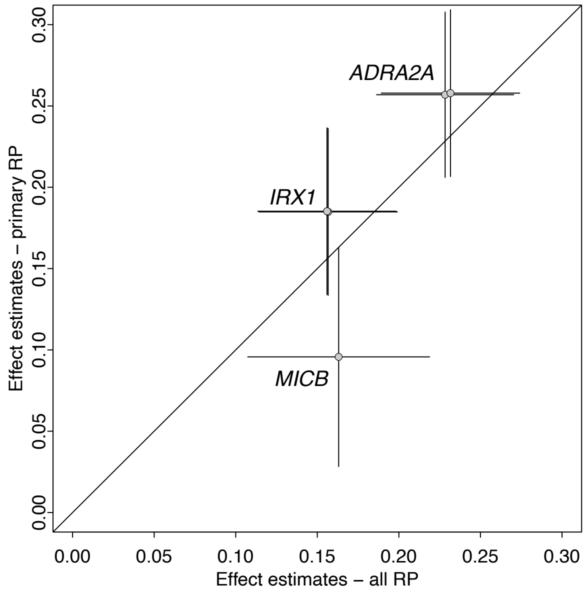

Supplementary Figure 4 Comparison of effect estimates for three genetic loci, five distinct variants, significantly associated with RP risk among all RP cases (x-axis) and when restricting to primary RP cases only (y-axis).

Supplementary Figure 5 – Genetic correlation plot

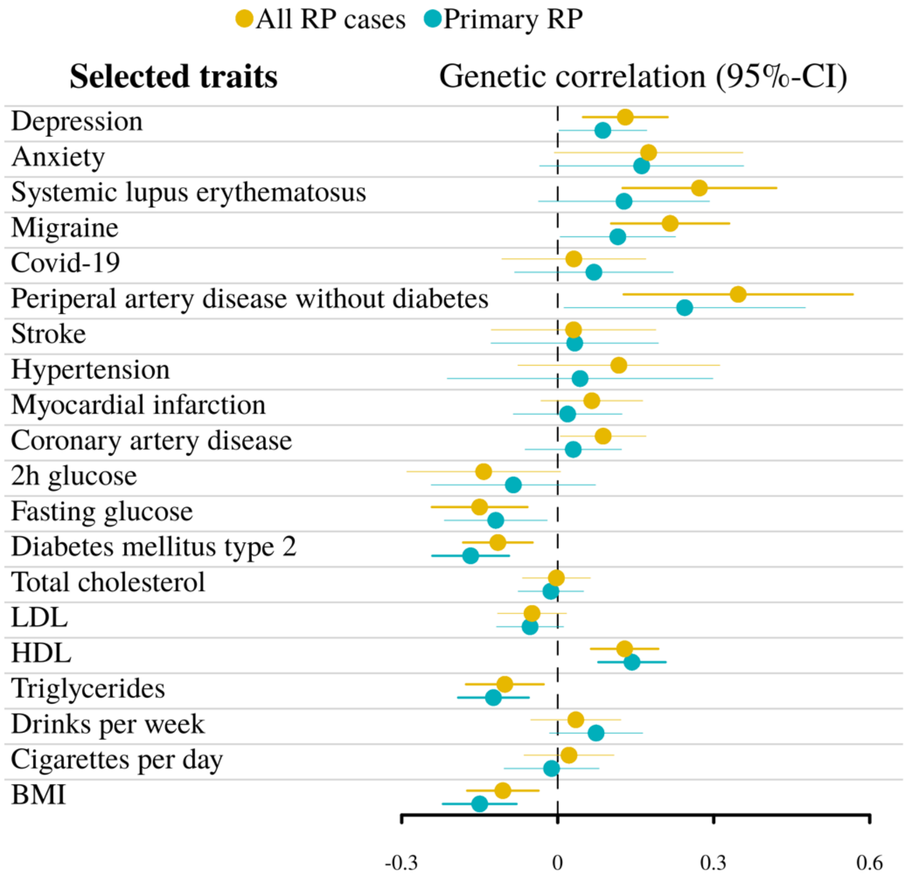

Supplementary Figure 5 Forest plot summarizing genetic correlation analyses between RP and selected traits Results for selected traits as listed in Supplementary Data 2. Dots indicate point estimates for genetic correlations and lines indicate 95%-confidence intervals.

Supplementary Tables

Supplementary Table 1 – Characteristics of cases and controls

Supplementary Tab. 1: Characteristics of case and control population Sample size, demographic factors, and comorbidities of Raynaud's phenomenon (RP) cases and controls. P values were obtained using Chi-Square test for categorical variables and ANOVA for continuous variables. All p-values are two-sided.

Variable (cases/controls (in case participants did not declare an answer))	***Participants with Raynaud's phenomenon***	***Controls***	***p-value***
n	5,147	439,294	
Age in years (mean (SD))	57.84 (7.9)	56.80 (8.0)	5.9×10^{-7}
Sex (%) [Male]	1,639 (31.8)	201,704(45.9)	$<2.2 \times 10^{-16}$
BMI in m2/kg (mean (SD))	25.86 (4.6)	27.42(4.8)	$<2.2 \times 10^{-16}$
Smoking (%) (9048/433817)			4.3×10^{-7}
Never	2,493 (50.6)	236,881 (54.1)	
Ever	2,535 (49.4)	200,856 (45.9)	
Alcohol (%)			6.5×10^{-4}
Never	205 (4.0)	13,789(3.1)	
Ever	4,937 (96.0)	425,114 (96.9)	
Diastolic blood pressure in mmHg (mean (SD))	80.49 (10.6)	82.22 (10.7)	$<2.2 \times 10^{-16}$
Systolic blood pressure in mmHg (mean (SD))	138.91 (20.2)	140.03 (19.6)	1.3×10^{-4}
Town Deprivation Index (mean (SD))	-1.42 (3.0)	-1.49 (3.0)	0.131
Household income in Pound (%)			$<2.2 \times 10^{-16}$
Less than 30,999	2,527 (49.3)	180,752 (41.3)	
31,000 to 100,000	1,658 (32.3)	177,331 (40.5)	
Greater than 100,000	116 (2.3)	20,216 (4.6)	
Not declared	827 (16.1)	59,498 (13.6)	
Migraine (%)	519 (10.1)	19,287 (4.4)	$<2.2 \times 10^{-16}$
Hypertension (%)	2,190 (42.5)	144,872 (33.0)	$<2.2 \times 10^{-16}$
Type 1 Diabetes mellitus (%)	60 (1.2)	4,058 (0.9)	8.4×10^{-2}
Type 2 Diabetes mellitus (%)	381 (7.4)	36,770 (8.4)	0.014
Other chronic ischemic heart disease (%)	601 (11.7)	29,892 (6.8)	$<2.2 \times 10^{-16}$
Systemic Lupus Erythematosus (%)	131 (2.5)	528 (0.1)	$<2.2 \times 10^{-16}$
Systemic Sclerosis (%)	229 (4.4)	148 (0.0)	$<2.2 \times 10^{-16}$

Supplementary Table 2 – OR for sex and sex*SNP interaction

Supplementary Tab. 2: OR for variants for men and women separatly as well as p-value for sex*SNP interaction term. Statistics were derived using logistic regression models.

SNP	Alleles	Frequency	GWAS	OR women	CI lower women	CI upper women	p-value women	OR men	CI lower men	CI upper men	p-value men	p-value interaction
rs7090046	A/G	0.31	all	1.28	1.21	1.36	1.01e-18	1.20	1.11	1.31	7.97e-06	0.20
rs12653958	A/G	0.30	all	1.14	1.07	1.20	8.58e-06	1.20	1.11	1.30	1.23e-05	0.28
rs3094013	G/A	0.14	all	1.15	1.07	1.24	1.24e_04	1.23	1.11	1.37	6.48e-05	0.31
rs1343449	A/G	0.32	primary	1.30	1.22	1.39	3.64e-14	1.19	1.09	1.30	1.71e-04	0.13
rs72731435	T/C	0.30	primary	1.17	1.09	1.26	9.39e-06	1.23	1.12	1.35	9.88e-06	0.41

OR = odds ratio; CI = confidence interval; GWAS = type of outcome, all – all RP cases, primary – excluding secondary RP cases

Supplementary Table 3 – Effect estimates for all and primary RP cases

Supplementary Tab. 3: Comparison of effect estimates for genetic variants associated with RP between all and primary cases. Statistics were derived from logistic regression models.

Variant					GWAS of all RP (N = 444,441, 5147 cases and 435,357 controls)			GWAS of Primary RP(N = 441,542, 3505 cases and 435,357 controls)		
SNP	Chromosome number	Position	Alleles	Frequency	OR	CI	P value	OR	CI	P value
rs12653958	5	4032849	A/G	0.30	1.17	(1.12;1.22)	4.76E-13	1.20	(1.14;1.27)	1.69E-12
rs72731435	5	4048526	T/C	0.30	1.17	(1.12;1.22)	5.85E-13	1.20	(1.14;1.27)	1.46E-12
rs3094013	6	31434366	G/A	0.14	1.18	(1.11;1.24)	9.73E-09	1.10	(1.03;1.18)	5.40E-03
rs1343449	10	112860526	A/G	0.32	1.26	(1.20;1.31)	1.85E-26	1.29	(1.23;1.36)	4.04E-23
rs7090046	10	112860930	G/A	0.31	1.26	(1.21;1.32)	9.58E-27	1.29	(1.23;1.36)	6.39E-23

Supplementary Table 4 – Latent causal variable analysis

Results from latent causal variable analysis for phecodes with evidence of significant genetic correlations (rg)

T1	***Phecodes***	***rg***	***SE(rg)***	***p.fdr(rg)***	***GCP***	***SE(GCP)***	***p.fdr(GCP)***
All RP cases	Osteoporosis NOS	0.40	0.08	1.36E-04	0.72	0.19	1.58E-03
Primary RP	Osteoporosis NOS	0.35	0.09	7.93E-03	0.66	0.22	1.17E-02
