# Supplementary material for: ADRA2A and IRX1 are putative risk genes for Raynaud’s phenomenon
Source: Nat Commun. 2023 Oct 12;14:6156. doi: 10.1038/s41467-023-41876-5 (PMC10570309; doi:10.1038/s41467-023-41876-5)
Supplement: Supplementary file 3 — Description of Additional Supplementary Files [file 41467_2023_41876_MOESM3_ESM.pdf]

Description of Additional Supplementary Files

File Name: Supplementary Data 1
Description: Results from cis-eQTL mapping for regional sentinels for Raynaud's syndrome; Statistics for RP were derived from logistic regression models and statistics for gene expression derived from linear regression models.

File Name: Supplementary Data 2
Description: Sources of publicly available GWAS.

File Name: Supplementary Data 3
Description: Results from genetic correlation analysis using selected traits.

File Name: Supplementary Data 4
Description: Results from genetic correlation analysis across 185 phecodes with two-sided tests of significance.

File Name: Supplementary Data 5
Description: Summary of genetically prioritized drug targets and drugs in trials for Raynaud's phenomenon.

File Name: Supplementary Data 6
Description: Electronic health records codes for identification of used conditions and diseases.
