# Supplementary material for: ADRA2A and IRX1 are putative risk genes for Raynaud’s phenomenon
Source: Nat Commun. 2023 Oct 12;14:6156. doi: 10.1038/s41467-023-41876-5 (PMC10570309; doi:10.1038/s41467-023-41876-5)
Supplement: Supplementary file 5 — Reporting Summary [file 41467_2023_41876_MOESM5_ESM.pdf]

nature portfolio

Corresponding author(s): Maik Pietzner and Claudia Langenberg

Last updated by author(s): 8/14/2023

Reporting Summary

Nature Portfolio wishes to improve the reproducibility of the work that we publish. This form provides structure for consistency and transparency in reporting. For further information on Nature Portfolio policies, see our Editorial Policies and the Editorial Policy Checklist.

Statistics

For all statistical analyses, confirm that the following items are present in the figure legend, table legend, main text, or Methods section.

n/a	Confirmed	
☐	☒	The exact sample size (*n*) for each experimental group/condition, given as a discrete number and unit of measurement
☐	☒	A statement on whether measurements were taken from distinct samples or whether the same sample was measured repeatedly
☐	☒	The statistical test(s) used AND whether they are one- or two-sided<br>*Only common tests should be described solely by name; describe more complex techniques in the Methods section.*
☐	☒	A description of all covariates tested
☐	☒	A description of any assumptions or corrections, such as tests of normality and adjustment for multiple comparisons
☐	☒	A full description of the statistical parameters including central tendency (e.g. means) or other basic estimates (e.g. regression coefficient) AND variation (e.g. standard deviation) or associated estimates of uncertainty (e.g. confidence intervals)
☐	☒	For null hypothesis testing, the test statistic (e.g. *F*, *t*, *r*) with confidence intervals, effect sizes, degrees of freedom and *P* value noted<br>*Give P values as exact values whenever suitable.*
☐	☒	For Bayesian analysis, information on the choice of priors and Markov chain Monte Carlo settings
☒	☐	For hierarchical and complex designs, identification of the appropriate level for tests and full reporting of outcomes
☐	☒	Estimates of effect sizes (e.g. Cohen's *d*, Pearson's *r*), indicating how they were calculated

Our web collection on statistics for biologists contains articles on many of the points above.

Software and code

Policy information about availability of computer code

Data collection	No software was used for data collection.
Data analysis	R (v4.1.2); REGENIE (v2.2.4); LDSC (v1.0.1); LDstore (v1.0); LCV (v.1.0); R packages susieR (v.0.11.92), liftOver (v.2.0), coloc (v.2.0), BEDtools (v1.5), PrediXcan (v.1.0)

For manuscripts utilizing custom algorithms or software that are central to the research but not yet described in published literature, software must be made available to editors and reviewers. We strongly encourage code deposition in a community repository (e.g. GitHub). See the Nature Portfolio guidelines for submitting code & software for further information.

Data

Policy information about availability of data

All manuscripts must include a data availability statement. This statement should provide the following information, where applicable:

- Accession codes, unique identifiers, or web links for publicly available datasets
- A description of any restrictions on data availability
- For clinical datasets or third party data, please ensure that the statement adheres to our policy

All individual level data is publicly available to bona fide researchers from the UK Biobank (https://www.ukbiobank.ac.uk/) or Genes & Health study (https://www.genesandhealth.org/). GWAS summary statistics have been deposited at the GWAS Catalog (accession codes: GCST90271713 and GCST90271714). Look-ups

and druggable targets were derived from the GWAS Catalog https://www.ebi.ac.uk/gwas/ and the OpenTargets Genetics data base (https://genetics.opentargets.org/).

Human research participants

Policy information about studies involving human research participants and Sex and Gender in Research.

Reporting on sex and gender	We included a total of 241,098 and 203,343 participants who self-identified as women and men, respectively, in our study. Out of 5147 participants diagnosed with Raynaud's phenomenon, 3508were women (see Supplementary Table 1). Given the sex-differential prevalence, we systematically tested all identified genome-wide significant loci for a potential sex-specific effects using logistic regression models with genetic variant by sex interaction terms.
Population characteristics	UK Biobank is a national resource that has been described extensively elsewhere (https://www.ukbiobank.ac.uk). Individuals were not directly selected for inclusion in the study on the basis of any disease or health parameter. Detailed demographics can be found in Supplementary Table 1.
Recruitment	All people aged 40–69 years (men and women) who were registered with the National Health Service and living up to 25 miles from one of the 22 study assessment centers were invited to participate in 2006–2010. Overall, about 9.2 million invitations were mailed to recruit 503,325 participants (a response rate of 5.47%).
Ethics oversight	All UK Biobank participants provided written informed consent, the study was approved by the National Research Ethics Service Committee North West–Haydock and all study procedures were performed in accordance with the World Medical Association Declaration of Helsinki ethical principles for medical research.

Note that full information on the approval of the study protocol must also be provided in the manuscript.

Field-specific reporting

Please select the one below that is the best fit for your research. If you are not sure, read the appropriate sections before making your selection.

☒ Life sciences ☐ Behavioural & social sciences ☐ Ecological, evolutionary & environmental sciences

For a reference copy of the document with all sections, see nature.com/documents/nr-reporting-summary-flat.pdf

Life sciences study design

All studies must disclose on these points even when the disclosure is negative.

Sample size	We used the full available sample in UK Biobank (with the exception of exclusions below) for discovery analyses. This is currently the largest sample size for patients with Raynaud's phenomenon that have also been genotyped.
Data exclusions	Individuals failing standard genotyping quality control parameters defined initially by the UK Biobank study or individuals of non-European ancestry were excluded from analysis. These decisions were made prior to performing any downstream analysis. In sensitivity analysis, we excluded participants who reported previous diseases or medications that might lead to RP as a secondary event.
Replication	We aimed for replication in the Genes & Health and BioVUstudy as described in the methods and main text, that replicated effects for the two main loci IRX1 and ADRA2A, respectively.
Randomization	N/A - randomization occurred naturally as genetic variants were the exposure.
Blinding	N/A - genetic association testing does not require blinding.

Reporting for specific materials, systems and methods

We require information from authors about some types of materials, experimental systems and methods used in many studies. Here, indicate whether each material, system or method listed is relevant to your study. If you are not sure if a list item applies to your research, read the appropriate section before selecting a response.

Materials & experimental systems

n/a	Involved in the study	
☒	☐	Antibodies
☒	☐	Eukaryotic cell lines
☒	☐	Palaeontology and archaeology
☒	☐	Animals and other organisms
☒	☐	Clinical data
☒	☐	Dual use research of concern

Methods

n/a	Involved in the study	
☒	☐	ChIP-seq
☒	☐	Flow cytometry
☒	☐	MRI-based neuroimaging
